# Is mental health staff training in de-escalation techniques effective in reducing violent incidents in forensic psychiatric settings? – A systematic review of the literature

**DOI:** 10.1186/s12888-023-04714-y

**Published:** 2023-04-12

**Authors:** Daniel Brenig, Pauline Gade, Birgit Voellm

**Affiliations:** Klinik für Forensische Psychiatrie, Universitätsmedizin Rostock, Rostock, Germany

**Keywords:** De-escalation, Staff training, Forensic psychiatry, Inpatient violence

## Abstract

**Background:**

Inpatient violence is a relevant issue in forensic psychiatric settings. Relevant guidelines recommend that restrictive measures are to be used exclusively if de-escalation and other preventive strategies have failed and there is a risk of harm to patients or staff if no action is taken. However, restrictive measures are untherapeutic and can be harmful. In order to enable staff to intervene before inpatient violence or other serious incidents occur and thus to avoid restrictive measures, mental health staff training programs including de-escalation components are being adopted in general as well as forensic mental health settings. There is growing evidence for the efficacy of mental health staff training in de-escalation techniques in the field of general psychiatry. However, there are no reviews evaluating the effectiveness of these interventions in reducing violent incidents in forensic psychiatric settings. Here we present the first literature review on the effectiveness staff training in de-escalation techniques in the field of forensic psychiatry.

**Method:**

We searched relevant databases for original research on the effectiveness of reducing violence in forensic psychiatric settings. Studies were included if they investigated staff training programs with de-escalation techniques in forensic mental health settings.

**Results:**

A total of 5 relevant studies were identified. None of the studies was a randomized controlled trial. Four studies were before and after comparisons without control group. A one group post-test-only design was used in one study. Methodological quality was low. The maximum sample size was 112 participants. Results indicated no relevant impact of mental health staff training in de-escalation techniques on the rate of violent incidents in forensic psychiatric wards. However, staff seemed to feel safer following the training. Results have to be interpreted cautiously due to several methodological and content-related limitations.

**Discussion:**

Evidence for the effectiveness of staff training in de-escalation techniques on reducing verbal and physical aggression in forensic settings remains very limited. The existing definitions of terms like de-escalation, de-escalation training and de-escalation techniques in the healthcare context appear rather vague. Although some positive changes are reported across a variety of outcome measures it remains unclear to what extent staff training in de-esclation techniques contributes to a reduction in aggressive incidents and restrictive measures in forensic psychiatry. The clinical implications of this review are therefore limited. Yet, an important implication for future research is that a more comprehensive approach might prove worthwhile. Conducting a further review integrating a wide range of complex interventions aimed at the reduction of inpatient violence rather than focusing on de-escalation only, might be a worthwhile approach.

**Supplementary Information:**

The online version contains supplementary material available at 10.1186/s12888-023-04714-y.

## Introduction

### Violence in general and forensic psychiatric settings

In mental health services violence is a current and relevant problem for professionals as well as patients [1]. A meta-analysis of 35 international studies including 23,972 inpatients showed that the proportion of patients who committed at least one act of interpersonal violence was 17% [2]. In a recent German study, including 64,367 admissions in psychiatric hospitals, 17,599 aggressive incidents were recorded throughout the year 2019 [3]. This study described that 5084 (7.90%) of the admitted patients showed aggressive behavior towards others. Amongst the 1,660 forensic inpatients included in this study, the proportion of aggressive behavior was even higher (20.54%). At least in Germany, data also suggest an increase of violent incidents in psychiatric hospitals over the last ten years [3].

British authors found that on forensic psychiatric wards there are higher rates of violence compared to general psychiatry [4, 5]. Referring to German psychiatric hospitals, forensic psychiatry had the highest proportion of cases with aggressive behavior (20.54%), but the number of incidents per bed was lower than in general adult psychiatry as well as in child and adolescent psychiatry [3]. Violent behaviour includes verbal and physical threats and aggression that may lead to serious injury or death. The risk of these behaviours is significant in forensic settings. This is due to the complex historical and current psychosocial needs of the patient group [6]. Among other things, the resulting damage includes physical and psychological injuries to fellow patients and staff, diminished therapeutic relationships, lower job-satisfaction of the employees as well as an increase in the number of days of sick leave [7–10].

### Restrictive interventions in general and forensic mental health services

Although they are untherapeutic, restrictive interventions (i.e. manual restraint, mechanical restraint, seclusion or forced medication) are used in psychiatric hospitals as well as in forensic mental health services in order to manage aggressive behaviour. However, this kind of coercion should exclusively be used if de-escalation and other preventive strategies have failed and there is potential for harm to patients or employees if no action is taken [11]. The use of restrictive interventions is very problematic for patients, staff and organizations [12]. They diminish the therapeutic alliance between staff and patients. Patients experience restrictive interventions as dehumanizing, frightening, confusing and at times painful [13, 14]. Restrictive interventions are associated with anxiety and stress and can cause physical and psychological damage for both patients and staff [15, 16]. Both staff and patients might suffer injury [17]. Moreover, especially mechanical restraint or isolation can be highly traumatic. Occasionally, restrictive measures can not only result in serious physical harm, but even patient deaths (e.g. due to physical restraint) [18, 19].

### The concept of de-escalation and de-escalation techniques in the healthcare context

The recommended first-line response to potential violence and aggression in healthcare settings is de-escalation [11, 20, 21]. This means that, especially in forensic psychiatric settings, staff need to intervene before situations escalate to a level when there seems to be no other choice but using restrictive interventions to protect themselves as well as the health and lives of their other entrusted patients. Referring to Bowers et al., until 2011 there seemed to be no systematic description of the concept of de-escalation in the healthcare context [22]. Nowadays, terms like “de-escalation”, “de-escalation techniques” or “de-escalation training” seem to be defined rather vaguely, as well. Nevertheless, it isn’t possible to reasonably define the content of de-escalation trainings without clarifying these terms [22]. For the purpose of this review it is therefore necessary to make a serious attempt to establish a working definition of de-escalation techniques.

In 2012 Price and Baker strived to clarify what the term *“de-escalation techniques”* means in current literature [23]. Accordingly, de-escalation techniques are *“*a set of therapeutic interventions frequently used to prevent violence and aggression within mental health services” [23]. They describe that de-escalation techniques consist of several *“*key components*”*. In a thematic literature synthesis Price and Baker found seven themes describing these key components. These themes were related either to staff skills (characteristics of effective de-escalators; maintain personal control; verbal and non-verbal-skills) or the process of intervening (engaging with the patient; when to intervene; ensuring safe conditions for de-escalation and strategies for de-escalation). In 2014, based on the aforementioned description of de-escalation techniques, Price et al. conducted a systematic review about the learning and performance outcome of mental health staff training in de-escalation techniques for the management of violence and aggression [24]. In this review they describe that de-escalation techniques aim to stop the escalation of aggression to either violence or the use of physically restrictive practices via a range of psychosocial techniques. These psychosocial techniques would *“*typically involve the use of non-provocative verbal and non-verbal clinician communication to negotiate a mutually agreeable solution to the aggressor’s concerns*”* [23, 24]. Referring to Price and Baker [23] Bower developed a simplified and rather linear model portraying *“*de-escalation as a process, starting with delimiting the situation, then moving on to clarification of the problem with the patient concerned, followed by reaching a resolution” [4]. According to Bower this process “is only likely to succeed if, at every stage, the de-escalator is controlling their own emotions and expressing respect and empathy for the patient they are seeking to de-escalate “. In 2017, Hallett et al. conducted a concept analysis of de-escalation of aggressive behaviour in healthcare settings [20]. They found that, considering the available literature, de-escalation in healthcare settings could be characterized as *“*a collective term for a range of interwoven staff delivered components comprising verbal and non-verbal communication, self-regulation, assessment, actions, and safety maintenance, which aims to extinguish or reduce or reduce patient aggression/agitation irrespective of its cause, and improve staff-patient relationships while eliminating or minimising coercion or restriction.” They also describe that de-escalation “comprises a set of skills, knowledge, and personal features in the domains of communication, self-regulation, assessment, activity, and safety maintenance “. Evidently, there is a great overlap in the aforementioned definitions regarding their key elements in terms of content. However, Hallet et al. as well as Baker use the term “de-escalation”, while Price and Baker refer to “de-escalation techniques”.

The current review focuses on the effectiveness of mental health staff training in de-escalation techniques in forensic psychiatric settings. The working definition of “de-escalation techniques” underlying this manuscript corresponds essentially to the aforementioned concept as suggested by Price et al. [23, 24]. Accordingly, de-escalation techniques are regarded as a set of interwoven, (partially) learnable, non-physical, psychosocial techniques with the aim of stopping an impending escalation of inpatient aggression to violence in mental health services. On the one hand, the key components of these de-escalation techniques include themes relating broadly to staff skills. These staff skills include verbal skills (e.g. negotiating, tactful language, using a calm tone of voice, sensitive use of humour), non verbal-skills (e.g. attentive posture and body language, active listening, a certain degree of eye contact), the ability to maintain personal control when faced with inpatient aggression as well as the ability to express a positive, emphatetic, supportive and non authoritarian therapeutic attitude. On the other hand, de-escalation techniques accordingly include themes relating broadly to the process of intervening. This implies the ability to engage with the patient and to make reasonable assessments (e.g. about the necessity and timing of intervening; about what level of staff support is necessary and whether the area is safe). Furthermore, de-escalation strategies are regarded as key components of de-escalation techniques (e.g. shared problem solving, facilitating expression, offering alternatives to aggression, limit-setting).

### Mental health staff training in de-escalation techniques within the field of general psychiatry

Training in de-escalation techniques is often a key feature of complex interventions for reducing restraint and seclusion [23]. For years, staff training including de-escalation components to prevent and reduce verbal and physical aggression has been adopted in mental health settings. These training programs intend to promote prevention, relational security and the de-escalation of conflicts. Several different training programmes are already in use.

A concrete example of such a training program is “ProDeMa” (Professional Deescalation Management) [25, 26]. ProDeMa is program that, referring to its authors, is explicitely focused on training a mental health staff on de-escalation techniques. The program was developed in Germany and intends to reduce violent incidents through 7 “de-escalation levels”:

1. Prevention/Reduction of violence through improvements concerning external framework conditions, e.g. aggression inducing ward rules or process flows
2. Change of reaction patterns of the staff through change in interpretation and valuation of inpatient violence
3. Improvement of the staff’s understanding of the etiology of violent behaviour
4. Training staff in verbal de-escalation techniques
5. Teaching staff techniques to escape and defend themselves against physical attacks without harming the patient unnecessarily
6. Techniques to immobilize and restrain patients without doing unnecessary harm to them
7. Professional post-processing of escalations including inter-collegial first aid.

All in all, in the field of general psychiatry the available data concerning key outcomes (e.g. assault rate, incidence of aggression, use of physical restraint) are rather mixed. Literature reviews about the effectiveness of de-escalation training respectively training in de-escalation techniques in reducing the use of coercive measures propose that more evidence is needed to evaluate their effectiveness [24, 27]. However, some weak indications for the efficacy of mental health staff training in de-escalation techniques in reducing violent incidents as well as the use of restraint and seclusion have already been found within the field of general psychiatry [24, 27, 28]. For example, a few studies found a significantly reduced risk of physical assaults on ward level [24, 29–31] respectively a significant reduction of aggressive incidents including verbal aggression and violence towards objects [24, 29, 31].

#### Implementation of menthal health staff training in de-escalation techniques in forensic psychiatric settings

Several authors call for the implementation of similar interventions in forensic psychiatric settings. For example, Bader and Evans state that in order to reduce inpatient violence, training for nursing staff would be as important as direct drug/medical treatment of patients [32]. Barr et al. assert that it is necessary for forensic nurses to develop de-escalation promoting, restrictive practices reducing and recovery-focused care promoting skills [33]. Maguire and colleagues also promote staff training with de-escalation techniques components [34]. Dexter and Vitacco note that, amongst other effective treatment interventions, aggression- and de-escalation training for staff should be implemented in order to prevent violence in forensic hospitals [35]. Goodman et al. precise that successful de-escalation in a high-secure forensic setting needs strong therapeutic relationships and knowledge about the relationship between trauma and aggression [36]. Given the adverse effects of coercive measures on patients, staff and organizations, it seems to be crucial that more evidence in this field is collected and analyzed.

Consequently, mental health staff training in de-escalation techniques is being implemented within the field of forensic psychiatry. Yet, there seems to be a crucial lack around training effectiveness. Therefore in this systematic review we will endeavor to present the current evidence for mental health staff training in de-escalation techniques in reducing violent incidents in forensic psychiatric settings.

## Method

In conducting this review we have followed the PRISMA guidelines for reporting systematic reviews [37].

### Search strategy

We conducted a systematic literature search of publications from 2002 (the year ProDeMa was developed) up until December 2021.The search included the electronic data bases Cochrane Library, Ovid PsycInfo, PubMed, Science direct, Scopus and Web of Science. We combined search terms capturing forensic settings with various terms relating to health care professionals as well as deescalation. The full search strategy is included in the Supplementary Material.

### In- and exclusion criteria

Our selection included studies related to the evaluation/assessment of a staff training program to reduce violent incidents in forensic psychiatric hospitals. Particular emphasis was placed on mental health staff training referring to de-escalation techniques regarding the research question "Is mental health staff training in de-escalation techniques effective in reducing violent incidents in forensic psychiatric settings?".

#### Inclusion criteria

Studies of any type of design were included if they met the following criteria:

- Original research
- Studies in which staff training with a de-escalation techniques component was investigated
- Studies conducted in forensic mental health settings
- Human participants of all ages in forensic mental health settings
- Male and/or female participants
- Any number of participants
- Studies in all languages and from all countries

#### Exclusion criteria

- Conducted in general psychiatric hospitals
- Training without de-escalation elements or attitudinal component
- Non-primary research, i.e. reviews, opinions, discussion papers

## Results

### Search results

**Fig. 1:**
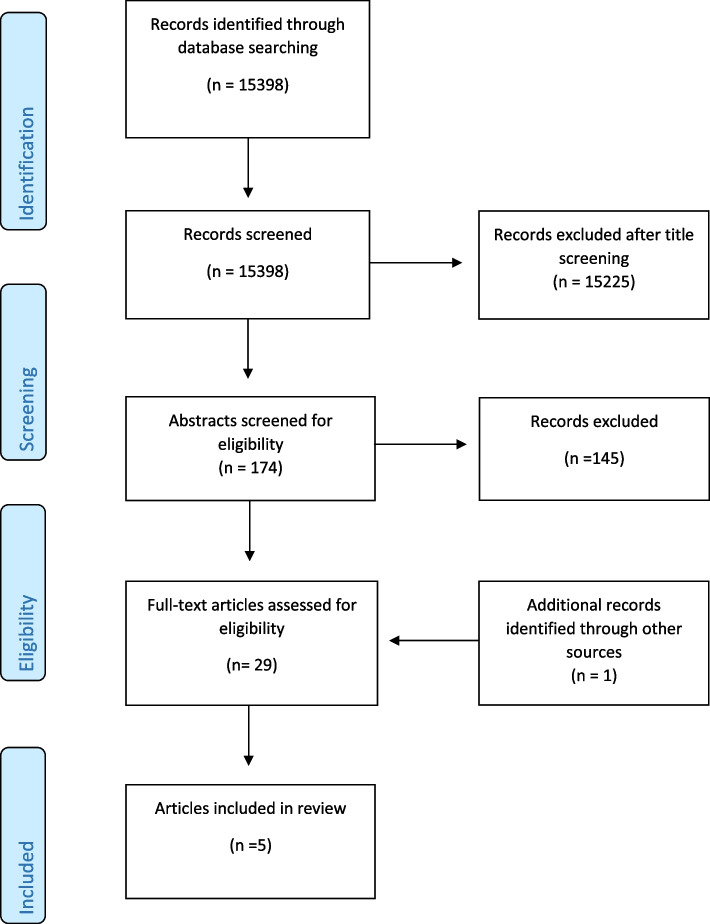
Flow of literature search results

The initial searches returned 15398 potentially relevant titles. Results of the searches were reviewed independently by authors PG and DB for suitability for inclusion in the review against the criteria set out below. This was initially undertaken through inspection of titles and abstracts. A second review appraising the full papers was then undertaken as required. In the event of a difference of opinion over a paper’s suitability for inclusion a third author (BV) was consulted. Additionally, authors DB and PG searched reference lists from both included and excluded studies for further suitable papers for inclusion. Using this approach, one more suitable study was found. A total of 174 papers were shortlisted because they seemed to describe studies in a forensic psychiatric hospital having deescalation training as a topic or being about describing the type, severity, frequency or reduction of violent incidents. After screening the abstracts 145 of these papers were excluded because they didn’t fit our selection criteria. Thirty full texts were finally screened. 5 papers fulfilled our selection criteria and were consequently included in this review. A flow chart of our search results is set out in Fig. 1. Details of the studies are shown in Table 1.

**Table 1:** Study findings

References	Study type	Country / type of unit / Location	Participants	Intervention	Outcome measures	Results
Isaak et al 2017 [38]	Before and after comparison without control group	Four forensic departments of a maximum security unit 132 beds Israel	112 employees (multiprofessional staff) *n* = 112 Return of questionnaires: 112 pre, 85 post training	Multiprofessional staff training including several de-escalation training elements (“Return home safely”); 3-day workshop	Number of aggressive or violent incidents Number of employees injured due to patient violence Occupational safety climate measured with the Safety Questionnaire (Mearns et al. 2003)	Significant decrease in number of aggressive incidents towards staff: 55 incidents in 2006 (pre intervention) vs 26 in 2008 and 13 in 2013 (post intervention) Significant decrease in number of employees injured: 36 in 2007 (pre) vs 24 in 2008/ and 12 in 2009 (post)
Isaak et al. 2018 [39]	Before and after comparison without control group	Four forensic departments of a maximum security unit in a mental health center 132 beds Israel	Multiprofessional staff *n* = 112	Multiprofessional staff training (“Return home safely”); annual refresher sessions	Number of aggressive or violent incidents Number of employees injured due to patient violence (Number of absenteeism days)	Number of aggressive incidents towards staff remains low: 55 incidents in 2006 (pre intervention) vs 18 in 2009/ 8 in 2010/ 6 in 2015/ 16 in 2016/ 13 in 2017 (post intervention)
Nesset et al. 2009 [40]	Before and after comparison without control group	Forensic psychiatric hospital 16 beds Norway	Nursing staff: *n* = 49 (rating 1); *n* = 48 (rating 2); *n* = 50 (rating 3) Patients: *n* = 10 (rating 1) *n* = 8 (rating 2) *n* = 8 (rating 3)	3-weeks nursing staff training program including several de-escalation training elements and lectures on milieu therapy	Self-report questionnaire WAS-R (revised Ward Atmosphere Rating Scale) to measure patients’ and staff’s experience of the treatment environment (Experience of involvement, support, practical orientation, angry and aggressive behavior, order and organization, staff control)	Significant decrease in WAS- subscale “Angry and aggressive behavior” (patients and staff)
Davies et al. 2016 [38]	Before and after comparison without control group	Medium secure forensic mental health service United Kingdom	79 staff members	Staff training in positive behavioral support (PBS)	Confidence in Coping with Patient Aggression Instrument (adapted version) Challenging Behaviour Attributions Scale Causal Dimension Scale II	Significant increase in staff’s confidence in their ability to manage challenging patient behavior following PBS-training
Martin and Daffern 2006 [41]	One group- posttest only-study	Secure forensic psychiatric clinic 100 beds Australia	69 clinicians	2-day workshop and refresher sessions of a staff aggression management/de-escalation training program (M4)	Adaptation of the Confidence in Coping With Patient Aggression Instrument (Thackrey 1987)	Increase in staff confidence in dealing with aggressive patients following training

### Description of study findings

The number of studies examining the effects of staff training in de-escalation techniques in forenic psychiatric settings was very limited. Five studies were finally deemed relevant for this review. The considered studies took place in hospitals in Israel (2), Norway (1), UK (1) and Australia (1). None of the studies was a RCT. One study was designed as a one-group-posttest-only. 4 studies were designed as before-and-after-comparisons-without-control-group. The number of participants per rating ranged from 8 to 112. The training periods lasted from 0,5 days to 3 weeks.

Nesset and colleagues conducted a pilot study in a Norwegian forensic psychiatric hospital consisting of 16 beds in order to investigate whether a nursing staff training program improves the ward atmosphere and patient satisfaction [40]. The 3 weeks staff training taught issues around de-escalation techniques using lectures as well as role plays. Week one focused on principles of milieu therapy, week 2 on how the nature of work in forensic psychiatry affects the nursing staff emotionally and how the staff could contain aggressive feelings from the patients. In week 3 setting limits was practiced, e. g. in role plays. After the intervention, nursing staff received no further teaching but weekly supervision continued and themes from the staff training program became a common element in these supervisions. The perception of the treatment atmosphere was measured by the revised Ward Atmosphere Scale (WAS-R) at three time points: before, immediately after and six months after the intervention [42]. The WAS-R is a self-report questionnaire including 11 subscales of which one measures the perception of angry and aggressive behavior displayed by the patients. Patients and staff reported a significantly lower level in the WAS-subscale “angry and aggressive behavior” after the intervention. The authors concluded that it might be possible to effectively improve the ward atmosphere through conducting a nursing staff training program [40]. However, besides the small sample size and the absence of a control group an important limitation of this study is that it didn’t evaluate explicitely whether the frequency (as opposed to the subjective assessment of patients and staff) of violent incidents actually reduced.

Martin and Daffern conducted a one-group-post-test-only study evaluating clinician perceptions of personal safety and confidence to manage inpatient aggression in a forensic psychiatric setting [41] following a staff training programme with a de-escalation techniques component, called M4 (“Managing the team, Managing the environment, Managing the patient and Managing aggression”). M4 consists of a 2-day workshop including theoretical (“organizational incidence and patterns of aggression, risk assessment, legal framework, therapeutic culture, crisis communication and deescalation skills, pharmacology, therapeutic interventions, critical incident stress management”) and practical elements (“self-defense and constraint”). All newly-appointed clinicians had to attend the workshop. After that, they were obliged to attend at least three refresher sessions (1,5 h each) per year. The main measured outcome results of the study were clinician perceptions of personal safety and confidence to manage inpatient aggression. These parameters were measured using a self-report questionnaire based on Thackrey’s “Confidence in Coping with Patient Aggression Instrument” [41, 43]. Clinicians reported the hospital as safe and found themselves relatively confident concerning their ability to manage aggressive patient behavior. Besides this, staff training on aggression management was reported as the most supportive factor on confidence in managing aggression. Whether the clinicians confidence in management inpatient aggression as well as the percepted personal safety translate into an actual reduction in incidents can, however, not be concluded reliably from this study. Neither is it possible to determine whether this positive assessment was objectively related to the training programme. Other limitations of the study were the small sample size, the absence of a control group as well as employing a questionnaire that was not validated.

Davies and colleagues investigated the effectiveness of multiprofessional staff training (79 trainees) in “positive behavioral support” (PBS) in increasing staff confidence and changing attributions of challenging inpatient behavior in a medium secure forensic mental health service in Wales, UK [44]. The training package around PBS included identifying primary and secondary (violence) prevention strategies. Methodologically the study was designed as a before and after comparison without control group. PBS includes de-escalation techniques such as verbal-deescalation and prevention of challenging behaviors. It can be described as a non-aversive approach to preventing and managing challenging behavior (e. g. aggressive/violent behavior of patients) through increasing the confidence of staff in their own abilities dealing with aggressive patients. Training for qualified staff took one day. It covered theoretical content as well as the practice of associated skills such as identifying primary and secondary prevention strategies of challenging behavior. Training for unqualified staff was limited to half-a-day and covered primarily theoretical aspects. To evaluate the effectiveness of the staff training program Davies et al. used self-report questionnaires. To measure the staff’s confidence an adapted version of Thackrey’s “Confidence in Coping with Patient Aggression Instrument” [43] was used. The staff's attributions of challenging inpatient behavior were measured using the “Challenging Behavior Attribution Scale” and the “Causal Dimension Scale “. After the intervention the confidence in working with challenging inpatient behavior increased significantly for both qualified and unqualified staff. Particularly for qualified staff, attribution of challenging behavior to external causes increased as well. It could be hypothesized that the staff’s confidence and attribution changes might have de-escalating effects and thus a violence reducing effect on the wards. However, an important limitation of the study is the fact that it didn’t focus on this concrete aspect. There is no evaluation whether the effects extend to objective data, such as numbers of actual incidents. Furthermore, there was no control group.

Isaak and colleagues examined the effectiveness of a 3-day intervention program (“Return home safely”) in a before and after comparison without control group in a high-secure forensic psychiatric (total of 132 beds) setting in Israel [38]. The training program was designed to enhance unit safety climate, to reduce patient violence and employee risk of injury from patient violence. The program contains several elements referring to the definition of de-escalation techniques as mentioned in the text above [23, 24]. Day one focusses on personal safety (i.e. how to avoid dangerous situations, self-defense skills, methods for safely restraining patients). Day 2 is mainly about tools for successful inter-staff communication. One day 3 staff issues around organizational learning are addressed (i.e. how to conduct incident investigations after adverse events). The outcome measures consisted of a questionnaire, recording of violent incidents and staff injuries. The 21-item safety climate questionnaire [45] distributed to hospital staff immediately before the workshop and again after 6 months contained 3 safety climate measures, i.e. communication about safety issues, procedures and safety reporting and perceived management commitment to safety. Following the training there was a significant improvement in perceived management commitment to safety as well as a marginally significant improvement in communication about safety issues as well as in procedures and safety reporting. The number of violent incidents and staff injuries also decreased significantly. Before the intervention program about 31 aggressive incidents toward staff were reported annually on average during the period from 2004 to 2007. After the intervention program about 15 aggressive incidents toward staff were reported annually on average during the period from 2008 to 2013. An important limitation of the study is its design, especially the absence of a control group.

The same group evaluated the (long-term) effectiveness of annual refresher training sessions of the training program at reducing critical incidents (e.g. physical aggression towards staff) [39]. The authors found that the rate of incidents in the years 2009 to 2017 was kept low in comparison to the pre-intervention years. About 12 aggressive incidents toward staff were reported annually on average during the period from 2009 to 2017. Again, important limitations of the study primarily are the small sample size and the absence of a control group.

## Discussion

This is the first systematic literature review examining the effectiveness of mental health staff training in de-escalation techniques in reducing violent incidents in forensic psychiatric settings. Unfortunately, inter alia due to the small number of relevant studies and their methodological weaknesses, only tentative conclusions can be drawn. The evidence base concerning the effectiveness of mental health staff training in de-escalation techniques in reducing violent incidents in forensic hospitals turned out to be poor.

Despite employing an extensive search strategy, in the field of forensic psychiatry we only found 5 relevant studies meeting our inclusion criteria. The studies were methodologically rather weak, not employing a randomized controlled design. Reliance on before and after comparisons without a control group limits the confidence in the reported findings, e. g. of the differences between trained and untrained groups [46]. In addition, the number of participants was quite small with a range from 8 to 112 participants. Only 2 of the 6 studies [43, 45 used “key safety outcomes” [24] such as rates or severity of violence, aggression, injuries or physical restraint. Two studies reported a significantly reduced number of aggressive incidents towards staff as well as a reduced number of employees injured after the staff training intervention [38, 39]. The remaining 3 studies found indirect indications for the effectiveness of staff training in de-escalation techniques in reducing violent incidents in forensic psychiatric settings, such as a lower level of perceived aggressive inpatient behavior [40], a significant increase of the staff's confidence in working with challenging inpatient behavior [44], or an increase in confidence in dealing with aggressive patients as well as in the perception of safety [41]. These studies mainly relied on surveys focusing on self-reported measures regarding the ability to de-escalate situations or the subjective perception of aggressive behavior on the wards. Whether this would also translate into effects on actual behavior and a concrete reduction in the number of violent incidents in those settings remains unclear.

We found a noteworthy variation across training programs in terms of topics covered as well as a considerably different number of training days (dosage). This makes it difficult to generalize findings across different studies employing different training programs. Participants might tend to answer surveys in the direction of social desirability causing overestimates in the positive effects of training on domains assessed through trainees’ self-reports [46, 47]. Only one study evaluated long term effects [39].

In conclusion, the findings of this review remain limited. Therefore only tentative conclusions can be drawn as to what extent de-escalation training leads to increased confidence in staff dealing with aggressive incidents and possibly even to a reduction in aggressive incidents in forensic psychiatry. However, from a clinical point of view it seems quite obvious that staff in forensic mental health settings need to be trained in de-escalation as this can be regarded as one of the core aspects of the profession. Future research on this topic seems to be absolutely necessary and should also focus on how this training can best be given in terms of time, form and content, i.e. which components should the training have and in which form and how often should it be delivered. Objective key outcomes like assaults on staff and other patients, injuries of staff and patients, inpatient verbal aggression and violence towards objects as well as use of physical restraints mustn ‘t be neglected. Subjective measures like job satisfaction and the subjective sense of security on the part of staff and inpatients should be evaluated, as well. Of course, it would be necessary to find out how long the effects last. Further research with observation as a method of data collection should especially focus on the effect of staff training in the level of objective and subjective competence of professionals as this probably reflects its most direct effect.

Noteworthy is that conducting this review it turned out to be quite demanding to find robust definitions for relevant terms like de-escalation, de-escalation training and de-escalation techniques. The preexisting definitions are questionable. The current review adopted the definition of “de-escalation techniques” in accordance with Price and Baker [23]. However, some doubts might arise whether the given definition of de-escalation techniques reflects the complexity of the relational and temporal context in which de-escalation is used. Compounding this problem is the fact that mental health staff training, even if they seem to focus on de-escalation or de-escalation techniques, usually consist of several other impact factors, as well. Even „ProDeMa “, a program predominantly focused on training a mental health staff on de-escalation techniques, apparently contains impact factors that go beyond the preexisting defintions of de-escalation (e.g. intercollegial first-aid).

In the field of general psychiatry, Hirsch et al. conducted a systematic review of the literature focusing on the efficacy of measures to avoid coercion in general [48]. Unlike Price et al., whose paper inspired the current review [24], Hirsch et al. didn’t limit their review to interventions including de-escalation components. They found that complex intervention programs seem to be particularly effective [48].

To conclude, for future research in the field of forensic psychiatry the limited outcome of this review with regards to clinical implications indicates that a more comprehensive approach might prove worthwhile. Aggression obviously occurs as a a result of a lot of different factors. This seems to make it difficult to find a convincing effect of one single variable, for example de-escalation skills of professionals. More precisely, conducting a further review dispensing with reference to de-escalation (techniques) and instead integrating a wide range of complex interventions (including “Safewards” and “Six Core Strategies”) aiming to reduce verbal and physical patient aggression as well as restrictive interventions might be an effective approach.

## Supplementary Information

**Additional file 1:** **Appendices.**

## Data Availability

The datasets used and/or analysed during the current study are available from the corresponding author on reasonable request.
