# Supplementary material for: Is mental health staff training in de-escalation techniques effective in reducing violent incidents in forensic psychiatric settings? – A systematic review of the literature
Source: BMC Psychiatry. 2023 Apr 12;23:246. doi: 10.1186/s12888-023-04714-y (PMC10099889; doi:10.1186/s12888-023-04714-y)
Supplement: Supplementary file 1 — Additional file 1: Appendices. [file 12888_2023_4714_MOESM1_ESM.docx]

**APPENDICES**

**Search strategy**

**PICO**

**P -** Population: Inpatients of forensic mental health settings, healthcare staff working within forensic mental health or correctional settings

**I - Intervention:** Studies in which a staff training with de-escalation techniques component was investigated in forensic psychiatric settings

**Comparator(s):** Not applicable

**Outcomes:** any

**Search terms:**

(health personnel OR health care worker* OR health provider* OR health care provider* OR healthcare provider* OR health care staff OR healthcare staff OR health care professional* OR healthcare professional* OR health staff OR health professional OR health worker OR medical staff OR medical worker OR medical provider OR medical personnel OR medical professional OR physician* OR doctor OR clinician* OR nurse* OR nursing staff* OR nursing assistant* OR practitioner* OR nurses’ aide* OR nursing aide* OR nurse’s aide* OR therapist* OR psychologist* OR allied health personnel OR allied health professional*) AND (forens* OR secur* OR crim* OR offend* OR justice OR judicia* OR court* OR invol* OR coerc* OR prison* OR impris* OR Gefängnis* OR jail* OR Haft OR Maßregelvollzug) AND (violen* OR aggress* OR angry OR hostil* OR assault*) AND (deescal* OR deeskal* OR de-escal* OR PRODEMA OR intervene* OR management OR train* OR professional development OR reduction OR decreas* OR program* OR contain* OR breakaway)

**Information sources**

Electronic Databases: Cochrane Central Register of Controlled Trials, Ovid PsycInfo, PubMed/MEDLINE, Science direct, Scopus, Web of Science

**Other sources:** searches of reference lists of identified papers, handsearching of relevant journals, conference programs, publication lists of relevant authors

**Others**

No language restrictions

Timeframe: 2002 onwards (year PRODEMA was developed)

**Number of search results and date of the last search:**

- Cochrane Library (Central Register of Controlled Trials) (1859 results, 28 November 2021)
- Ovid PsycInfo (228 results, 05 December 2021)
- PubMed/MEDLINE (7422 results, 30 November 2021)
- Scopus (1367 results, 01 December 2021)
- Web of Science (4522 results, 29 November 2021)
